# Using D-Dimer to Diagnose Painless Acute Aortic Dissection: A Case Report

**DOI:** 10.1155/2011/395613

**Published:** 2011-09-18

**Authors:** Caroline Barniol, Baptiste Vallé, Emilie Dehours, Sandrine Charpentier, Vincent Bounes, Dominique Lauque

**Affiliations:** Pôle de Médecine d'Urgence, Hôpitaux Universitaires de Toulouse, CHU Purpan, TSA 40031, 31059 Toulouse Cedex 9, France

## Abstract

*Introduction*. Aortic dissection is a cardiovascular emergency; the most frequent symptom is chest pain, but clinical presentation can be varied and atypical. *Case Presentation*. We report the case of a 66-year-old Caucasian male who presented a syncope immediately followed by a left-arm weakness while driving his car. Clinical examination was normal, but bilateral jugular vein distension was noted. Electrocardiogram and chest radiography were unremarkable. Among blood tests performed, troponin I test result was negative, and D-dimer test concentration was >4000 ng/mL. Since D-dimer test result was positive, chest computer tomography angiogram was performed and found a thoracic aortic dissection. *Conclusion*. Our case report shows that acute aortic dissection diagnosis is difficult and must be associated with the interpretation of various clinical signs and D-dimer measurement. It could be helpful for the emergency physician to have a pretest probability D-dimer like in pulmonary embolism diagnosis.

## 1. Introduction

Aortic dissection is a cardiovascular emergency; the most frequent symptom is a chest pain, but clinical presentation can be varied and atypical [1]. 

These atypical presentations often lead to a delayed diagnosis. Indeed, carrying out a scan confirming this diagnosis hypothesis of aortic dissection is late and may cause a delay of care. This period may be shortened by D-dimer test in the face of any faintness in ICU [2, 3]. We detail here an aortic dissection (AD) with atypical presentation for which D-dimers were immediately made. This allowed the early implementation of a scanner and rapid surgical supporting of the patient.

## 2. Case Presentation

**Figure 1:**
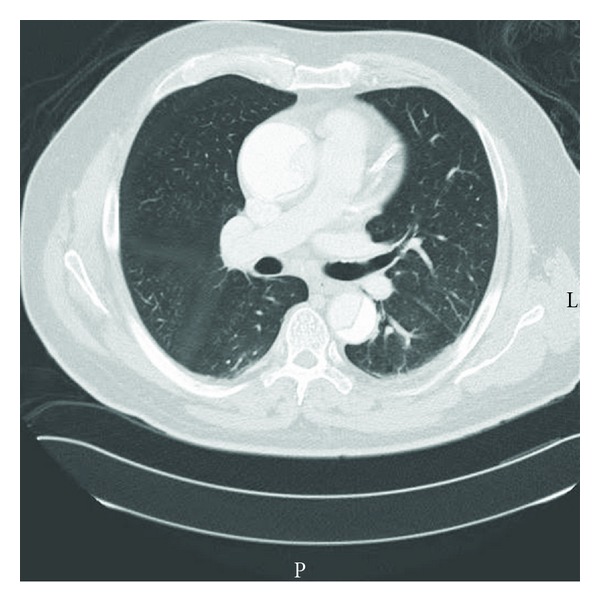
Axial computed tomography scan. Image shows thoracic aortic dissection (De Bakey/Standford type I/A).

A 66-years-old Caucasian man presented a syncope immediately followed by a left-arm weakness while driving his car. On scene, there were no accident, and the patient was conscious but mentally confused and had repetitive questioning. Blood pressure was 119/69 and 136/90 in the left and right arms, respectively, the pulse rate was 54 beats/min, regular, and the pulse oximetry was 100%. The patient had a past medical history of high blood pressure and was treated for hypercholesterolemia. In the emergency department, he had no pain, and mental confusion was improved, but a transient global amnesia was observed. Neurological examination was normal, and bilateral jugular vein distension was noted. Electrocardiogram and chest radiography were unremarkable. A transient ischemic attack or epilepsy was suspected. The computer tomography (CT) scan of the head did not reveal any abnormal finding. During observation, a convulsion occurred during 5 seconds. Concomitant pulse rate was 45/min and blood pressure 80/60 mmHg and 100/80 mmHg in the left and right arms, respectively. Among blood tests performed, troponin I test result was negative, and D-dimer Elisa test concentration was >4000 ng/mL (normal range <500 ng/mL). Since D-dimer test result was positive, chest CT angiogram (4 hours after the arrival) was performed and found a thoracic AD extending ascending aorta, aortic arch vessels, and descending aorta to superior mesenteric artery, without thrombus in false lumen nor pericardial effusion (Figure 1). This De Bakey/Standford type I/A AD was repaired with a bridge tube in ascending aorta. The patient recovered without complication and was discharged home in a good condition twenty days after surgery. 

## 3. Discussion

Time is critical for AD management and outcome, but diagnosis is often delayed when clinical presentation is atypical. Aortic dissection symptoms and signs are various and have low sensitivity. Neurological symptoms are observed only in one third of patients with aortic dissection type A and are not always associated with or followed by chest pain. Neurological symptoms appear when dissection reaches the proximal portion of the aortic arch. Propagation of the dissection and false lumen to the cerebral arteries could transiently reduce cerebral blood flow and entail an ischemic stroke with focal signs or an hypoxic encephalopathy with syncope, seizure, or global amnesia. In our case, neurological signs could also be explained by systemic hypotension and related cerebral perfusion deficit [4].

The sensitivty of D-dimer test for AD diagnosis is reported as close to 100% in the literature and has a negative predictive value between 92% and 100% [5, 6]. Two meta-analyses confirmed that D-dimer testing is useful for the diagnosis of suspected acute aortic dissection [2, 3]. D-dimer is a fibrin damage product released by the activation of the coagulation factors and thrombosis in the false lumen of aortic wall. High D-dimer concentration reflects anatomical extended disease and false lumen communication with aortic lumen and blood circulation. However, D-dimer does not enter blood stream when complete thrombosis is present [7, 8]. Recently, negative D-dimer test results were reported in short-length AD or in dissection with thrombus in false lumen [7]. Moreover, D-dimer test results vary with time. Suzuki et al. [8] reported that D-dimer levels decreased as soon as six hours after symptom onset of AD. In our case, D-dimer testing was early sampled two hours after onset symptoms, and the thoracic aortic dissection was extended without thrombus on CT scan. We do not check D-dimer for all patients. In our emergency department, D-dimer test is indicated for patients with dyspnea or chest pain.

Since AD is an infrequent disease that presents with various symptoms and signs, post-testing D-dimer probability cannot be calculated for clinical prediction rules as in other diseases such as pulmonary embolism [9] (in 45 minutes, the result of D-dimer test is obtained). Even if D-dimer test is a valuable addition test for AD diagnosis, it must be always completed by imaging [10].

## 4. Conclusion

A neurological symptom without chest pain is a rare clinical presentation of AD. D-dimer measurement is helpful for the physician to order imaging tests. For this patient, thoracic CT scan was performed, because D-dimer test was positive. Our case report shows that acute aortic dissection diagnosis is difficult and must be associated with the interpretation of various clinical signs and D-dimer measurement.

##  Authors' Contributions

C. Barniol was the medical physician in charge of our patient throughout his hospitalization and made substantial contributions to conception, acquisition, analysis, and interpretation of data and drafting the paper. B. Vallé and E. Dehours were involved in conception, design, interpretation and writing of the paper. S. Charpentier, V. Bounes, and D. Lauque were involved in revising the paper and final approval of the version. All authors have been involved in critically revising the paper for intellectual content, and they have read and approved the final version submitted.

##  Consent

Written informed consent was obtained from the patient for publication of this case report and any accompanying images. A copy of the written consent is available for review by the Editor-in-Chief of this journal.

##  Conflict of Interests

The authors declare that they have no conflict of interests.
